# Clinical and anatomical features of the lateral costal artery and vein

**DOI:** 10.1038/s41598-022-14318-3

**Published:** 2022-06-22

**Authors:** Seshiru Nakazawa, Natsuko Kawatani, Kai Obayashi, Yoichi Ohtaki, Tomokazu Ito, Toshiki Yajima, Ken Shirabe

**Affiliations:** 1 Department of General Surgical Science, Graduate School of Medicine, Gunma University, Maebashi, Japan; 2 Department of General Thoracic Surgery, Fukaya Red Cross Hospital, Fukaya, Japan; 3 Department of Innovative Cancer Immunotherapy, Graduate School of Medicine, Gunma University, 3-39-22 Showa-machi, Maebashi, 371-8511 Japan

**Keywords:** Anatomy, Classification and taxonomy

## Abstract

The lateral costal artery and vein are under recognized yet potentially important vessels for physicians, especially cardiothoracic surgeons. This study sought to determine the prevalence and clinical, anatomical, and radiological features of lateral costal vessels. We retrospectively analyzed lateral costal vessels based on intraoperative images in patients who underwent thoracic surgery at our institute between January 2016 and March 2020. Clinical data and surgical videos were analyzed for patient characteristics, prevalence, length, laterality, and additional anatomical and radiological features. The overall prevalence of lateral costal vessels was 19% and was significantly higher in males than females (22% vs. 14%, *p* = 0.003). The lateral costal vessels extended beyond the 2nd intercostal space in 74% of the cases, with differing length between the right and left sides in bilateral cases. Lateral costal vessels could be identified intraoperatively using indocyanine green or preoperatively through three-dimensional computed tomography. The prevalence of lateral costal vessels is relatively high and should be acknowledged by physicians prior to procedures involving the vessels.

## Introduction

The lateral costal artery (LCA) is a branch of the internal thoracic artery (ITA), which runs downwards, backwards, and laterally from the ITA^1^. The LCA is clinically important for cardiac and thoracic surgeons for several reasons. Firstly, cardiac surgeons should be aware that a well-developed LCA can cause a steal phenomenon after coronary artery bypass grafting (CABG), leading to angina pectoris, or acute coronary syndrome^2^. Furthermore, the LCA itself can eventually be used as a bypass conduit for CABG in some cases. Hartman et al. have performed CABG by using the ipsilateral ITA and LCA as grafts for the right coronary artery and diagonal branches^3^. Secondly, for thoracic surgeons, a well-developed LCA can interfere with pleurectomy or thoracotomy^4^. Physicians should also be aware of injury to the LCA, which could cause hemothorax after thoracentesis or in cases of trauma.

Although clinically important, the LCA has yet to be clearly defined, with varying data published among studies. Initial reports were based on cadaveric studies, potentially limiting the number of cases and extent of analysis^1^. Moreover, the diverse nomenclature of the LCA, such as lateral internal mammary/thoracic artery, accessory internal mammary/thoracic artery, lateral infracostal artery, or retrocostal artery, creates confusion^1,3^. This study sought to clarify the clinical and anatomical features of the LCA based on intraoperative images in a large cohort of > 800 patients, with additional focus on the lateral costal vein (LCV). Moreover, this study aimed to uncover additional features and methods for detecting lateral costal vessels, using indocyanine green (ICG) or three-dimensional (3D) reconstructed images.

## Results

### Patient and procedure characteristics

A total of 973 procedures were analyzed (Supplementary Fig. 1). We first excluded 148 cases in whom the lateral costal vessels could not be visualized due to patient-related (n = 78, adhesion of the lung or thick pleura) or procedure-related (n = 70; mediastinal-, lower lobe-, and empyema-related procedures; median sternotomy or thoracotomy approaches) reasons. We next excluded procedures performed on the same patient and/or the same side. To analyze background patient characteristics, we excluded 44 cases in which procedures were performed on a same patient. To analyze anatomical characteristics, we excluded 17 cases in which procedures were performed on the same patient and the same side. Table 1 summarizes the sex, age, and laterality according to presence of lateral costal vessels. The overall incidence of lateral costal vessels was 19%, with rates being significantly higher in male than female patients (22% vs. 14%, *p* = 0.003). The median age was 69, with no difference in incidence or length of lateral costal vessels according to age (*p* = 0.335 and 0.609, respectively; Supplementary Fig. 2). We analyzed 493 and 315 right- and left-sided cases, respectively, with no difference in laterality according to the presence of lateral costal vessels (*p* = 0.693).

**Table 1:** Patient characteristics.

	Total	Lateral costal vessel absent	Lateral costal vessel present	*p* value
**Characteristics of patients**				
Total	781	632 (81%)	149 (19%)	
Sex				0.003
Male	477	370 (78%)	107 (22%)	
Female	304	262 (86%)	42 (14%)	
Age				0.335
Median	69	69	69	
IQR	61–74	61–74	59–74	
**Characteristics of anatomy**				
Total	808	657 (81%)	151 (19%)	
Laterality				0.693
Right	493	403 (82%)	90 (18%)	
Left	315	254 (81%)	61 (19%)	

Patients (upper part) and laterality (lower part) were classified according to absence or presence of lateral costal vessels.

*IQR* interquartile range.

### Anatomical features

**Figure 1:**
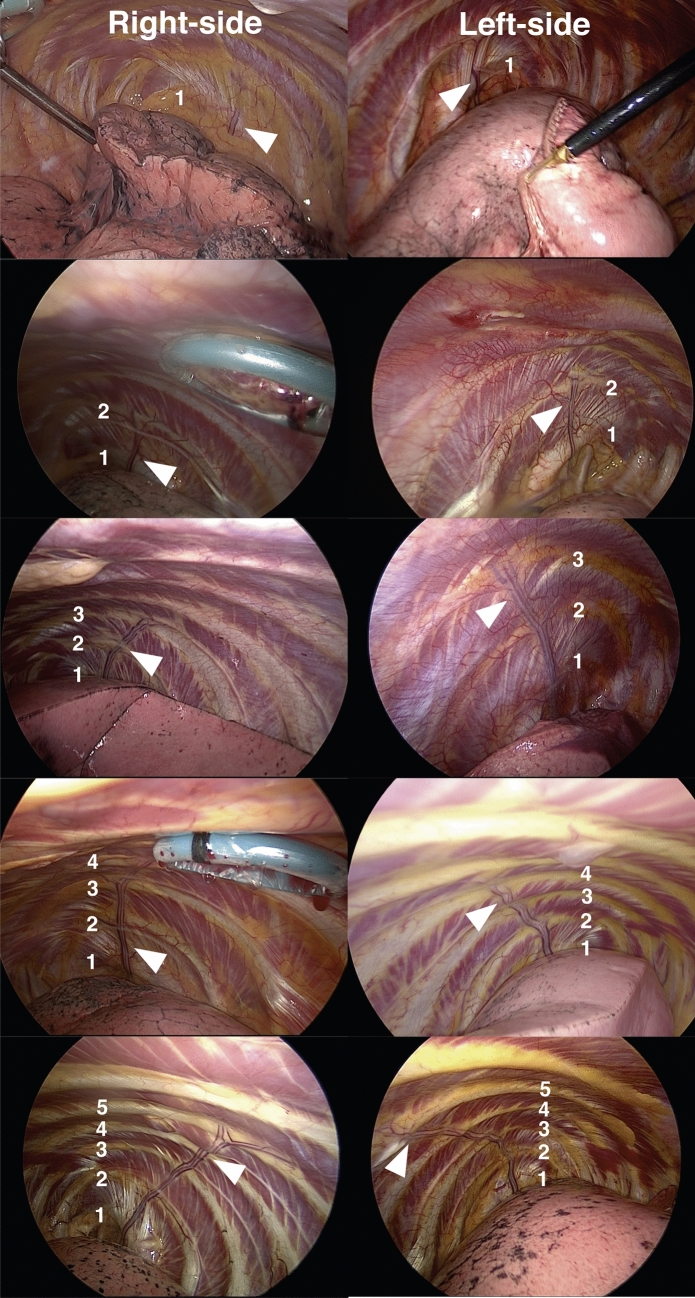
Intraoperative view of the lateral costal vessels. Representative cases of lateral costal vessels extending to the 1st, 2nd, 3rd, 4th, and 5th intercostal space, on right and left side (arrowheads). The ribs are numbered from 1 to 5.

**Figure 2:**
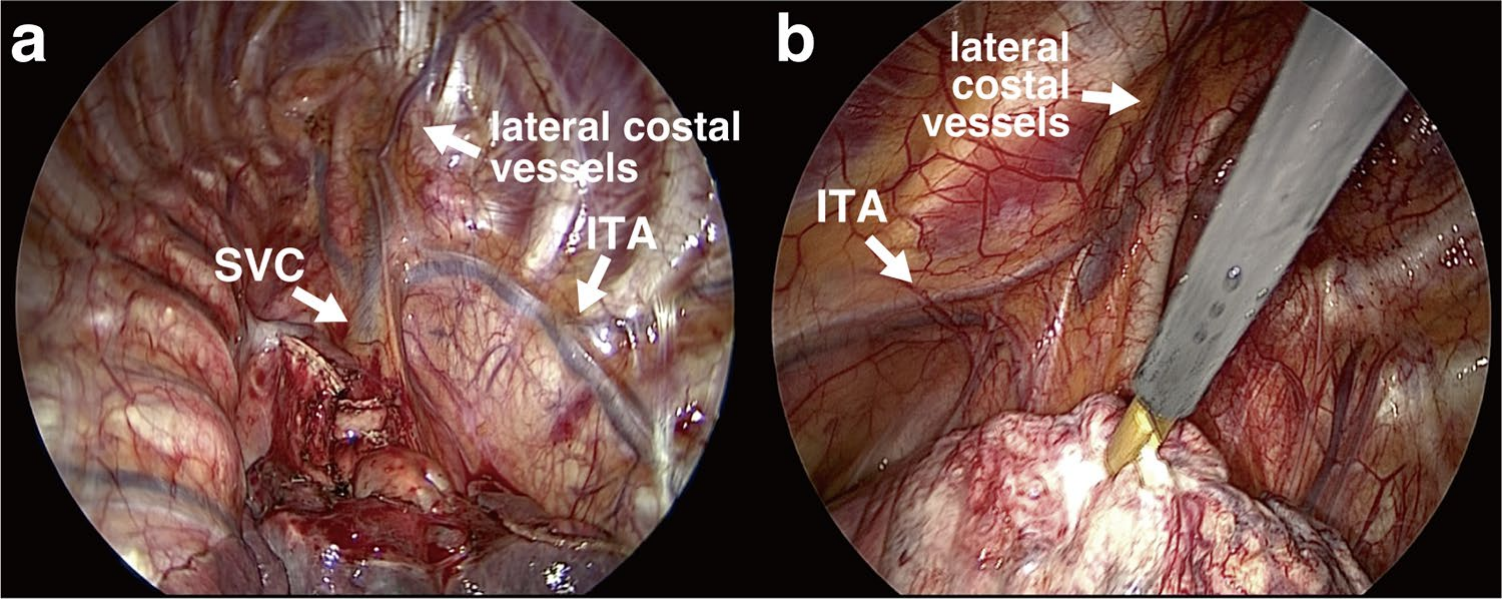
Branching point of the lateral costal vessels. Branching point of lateral costal vessels in relation to the superior vena cava and internal thoracic artery on the right (**a**) and left side (**b**). *SVC* superior vena cava, *ITA* internal thoracic artery.

**Figure 3:**
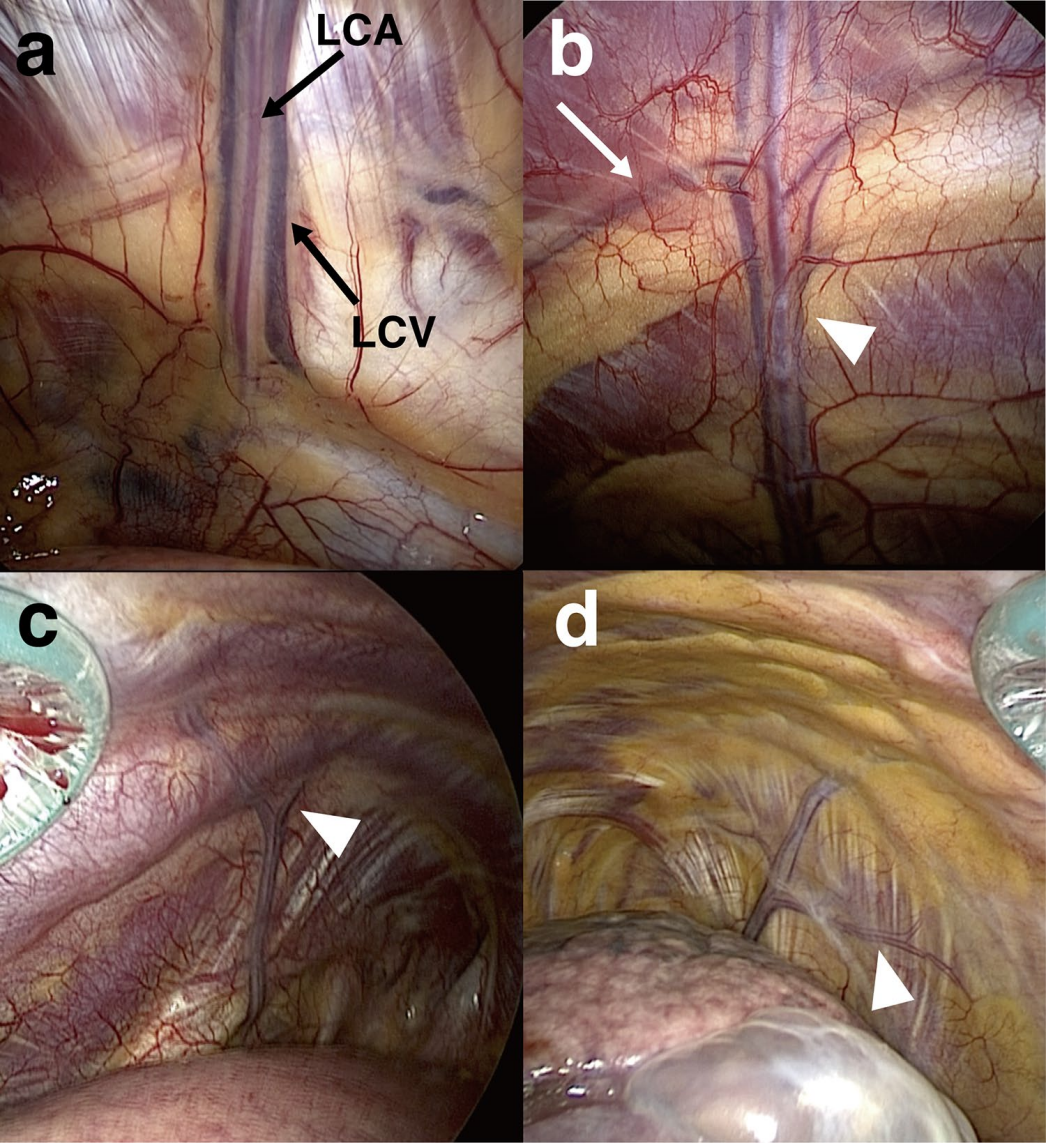
Anatomical features of lateral costal vessels. (**a**) The lateral costal artery (LCA) and concomitant lateral costal veins (LCV). (**b**) Anastomosis between the lateral costal vessels (arrowhead) and intercostal vessels (arrow). (**c**) Bifurcated-type left-sided lateral costal vessels (arrowhead shows bifurcation point). (**d**) Bifurcated-type right-sided lateral costal vessels (arrowhead shows bifurcated branch). *LCA* lateral costal artery, *LCV* lateral costal vein.

In most cases, the lateral costal vessels ended prior to the 5th rib (Fig. 1, Table 2). However, in 4% of cases, the vessels extended as far as the 5th intercostal space (ICS). The proximal part of the lateral costal vessels usually merged with the internal thoracic vessels (Fig. 2). When we observed the lateral costal vessels from a closer view, the LCA ran at the center, accompanied by concomitant LCVs (Fig. 3)^3^. We could also observe the anastomosis between the lateral costal vessels and intercostal vessels (Fig. 3b). Also, we occasionally encountered cases of bifurcated lateral costal vessels, which were either Y-typed or T-typed (Fig. 3c,d). Intraoperative data was available for both sides in 27 cases. Interestingly, only 59% of the cases had bilaterally missing lateral costal vessels, and lateral costal vessels were present unilaterally in 33% (right 22% and left 11%) and bilaterally in 7% of the cases (Table 3). Differences in length were observed in 2 cases whose lateral costal vessels were present bilaterally, with the right side being longer.

**Table 2:** Extent of lateral costal vessels.

Extent of lateral costal vessels	Total	Right	Left
	n = 151	n = 90	n = 61
1st ICS	39 (26%)	26 (29%)	13 (21%)
2nd ICS	43 (28%)	27 (30%)	16 (26%)
3rd ICS	35 (23%)	18 (20%)	17 (28%)
4th ICS	28 (19%)	14 (16%)	14 (23%)
5th ICS	6 (4%)	5 (6%)	1 (2%)

The lateral costal vessels were classified according to the intercostal space to which they extended.

*ICS* intercostal space.

**Table 3:** Cases of patients with bilateral intraoperative data.

Presence	n = 27	Extent of lateral costal vessels (ICS)	n
Bilateral	2 (7%)	Right 5th ICS and left 1st ICS	1
		Right 5th ICS and left 3rd ICS	1
Right side only	6 (22%)	1st ICS	2
		2nd ICS	3
		3rd ICS	1
Left side only	3 (11%)	1st ICS	1
		3rd ICS	2
Absent on both sides	16 (59%)	Not assessed	0

Patients with bilateral data were analyzed for presence and extent of lateral costal vessels.

*ICS* intercostal space.

### Lateral costal vessels detected with ICG and 3D-CT imaging

**Figure 4:**
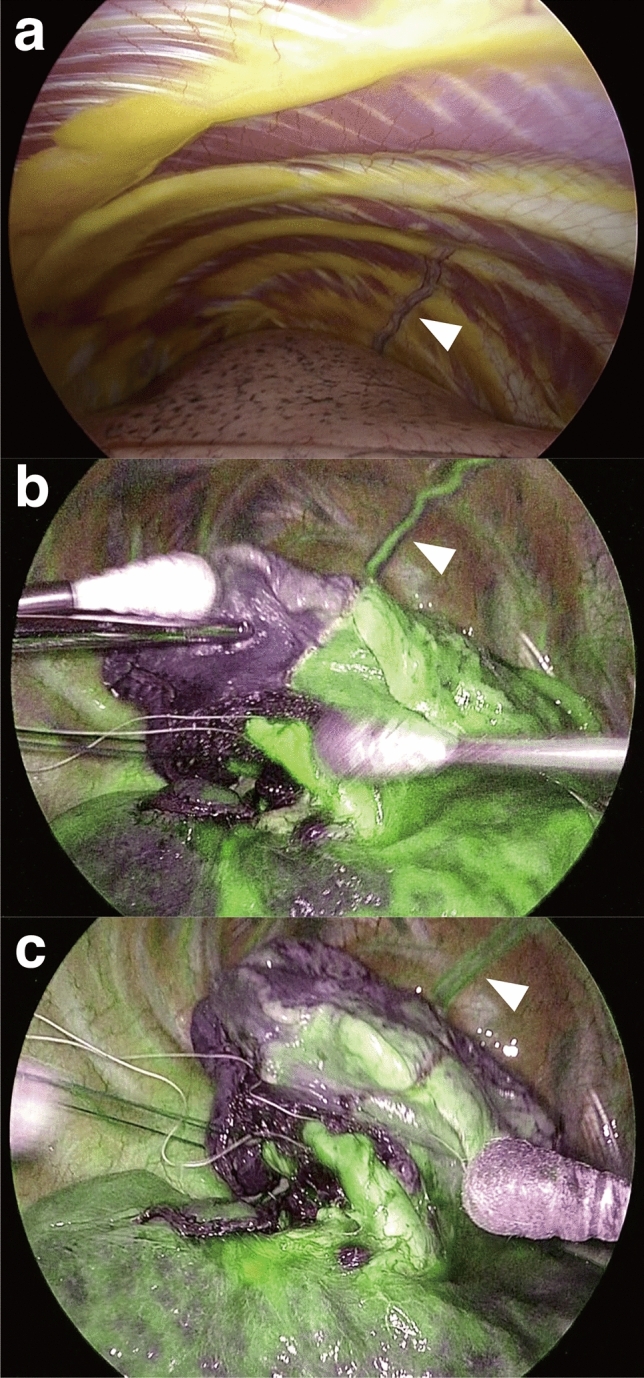
Lateral costal vessels depicted using indocyanine green. (**a**) View of the lateral costal vessels (arrowhead) prior to indocyanine green intravenous injection. (**b**) Initial detection of the lateral costal artery (arrowhead). (**c**) Subsequent detection of lateral costal veins (arrowhead).

**Figure 5:**
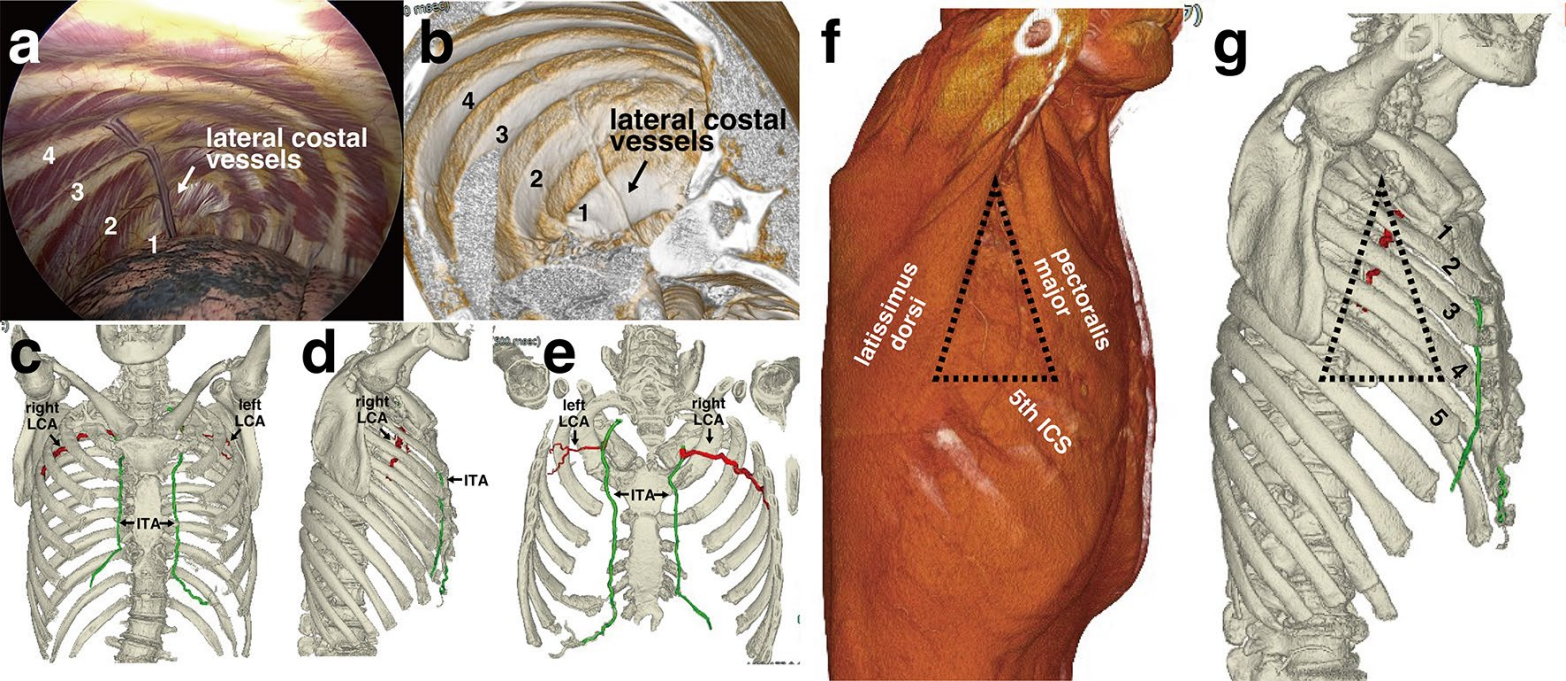
Three-dimensional images of lateral costal vessels. Comparison between the intraoperative view (**a**) and 3D-CT image (**b**) in a patient with left-side lateral costal vessels. The ribs are numbered from 1 to 4. The spatial position of lateral costal artery (LCA, red) and internal thoracic artery (ITA, green) in relation to the thoracic cage in a patient with bilateral lateral costal vessels. Frontal (**c**), lateral (**d**), and dorsal (**e**) image. Three-dimensional image of ‘triangle of safety’ (shown in black dotted line), bordered by the pectoralis major, latissimus dorsi, and 5th intercostal space (**f**). Relation between the ‘triangle of safety’ (black dotted line) and the course of lateral costal vessels extending to the 4th intercostal space (shown in red). The ribs are numbered from 1 to 5 (**g**). *LCA* lateral costal artery, *LCV* lateral costal vein, *ITA* internal thoracic artery, *ICS* intercostal space.

When we analyzed surgeries using intravenous injection of ICG as intraoperative guidance, we encountered several cases for which the lateral costal vessels were clearly depicted with ICG (Fig. 4). The LCA was enhanced first, followed by a delayed and weak enhancement of the LCV. Also, three-dimensional (3D) reconstruction of lateral costal vessels based on preoperative computed tomography (CT) data showed a good correlation between the intraoperative view and 3D-CT images (Fig. 5a,b). The 3D-CT images were especially useful to understand the spatial relationship between the LCA, the ITA, and the thoracic cage from different views (Fig. 5c–e), or in relation to the ‘triangle of safety’ (Fig. 5f,g).

## Discussion

This study retrospectively analyzed the LCA and LCV based on intraoperative thoracoscopic images, which allowed relatively good visual recognition. Our finding can be summarized as follows: (1) 19% of the included cases presented with lateral costal vessels, most of which extended further than the 2nd ICS, with differing length between the right and left sides; (2) prevalence was significantly higher in males than females; (3) lateral costal vessels can be running at the center of the ‘triangle of safety’, and can be confirmed by 3D-CT.

The incidence rate of LCA in the current study was 19% and was relatively similar to that presented in previous reports, which ranged from 10 to 30%^1,5–8^. Differences in incidences rate or correlation with gender among studies could have been attributed to the fact that initial reports on the LCA were mostly based on cadaveric studies, limiting the number of cases and extent of analysis. Other studies were also based on visual detection from the median sternotomy incision during cardiac surgery or during thoracoscopic procedures^4,7^. Furthermore, Houbois et al. recently analyzed the LCA based on contrast-enhanced CT or angiography^6^. The aforementioned study showed good inter-observer agreement for LCA detection, with a reported LCA incidence rate of 11%.

A characteristic feature of our study was that visual assessment of the LCA through thoracoscopy provided additional anatomical information, such as identification of concomitant LCVs, anastomosis with intercostal vessels, and bifurcated-type lateral costal vessels. The LCVs have rarely been focused, perhaps due to lower clinical relevance. We have recently introduced ICG during lung segmentectomy for the detection of the intersegmental plane. Our review found one surgical case wherein the lateral costal vessels were visualized using ICG. As shown in Fig. 5, the intraoperative images clearly showed the early enhancement of the LCA running at the center, in contrast to the delayed enhancement of the LCVs. Moreover, we have routinely used 3D-CT imaging for guidance during lung surgery^9^. After reviewing 3D-CT images of patients with lateral costal vessels, we were able to depict the lateral costal vessels through 3D-CT in patients with enhanced CT, which is relatively easy to use and allows an intuitive recognition. As described in the literature, the 3D-CT images also confirmed the course of lateral costal vessel, running laterally, downward, and backwards of the ITA, on the inner side of the midaxillary line^1^.

The LCA has been previously considered clinically important for patients who underwent CABG using the ITA as a bypass conduit^2,3^. Because the LCA often branches from the ITA, studies suggest that the LCA should be obliterated or dissected when performing CABG using the ITA as graft, in order to prevent steal phenomenon. Furthermore, Hartman et al. have reported that CABG using the LCA itself as a graft is also feasible^3^. In regard to the use of the LCA as a bypass conduit, our report showed that the length of LCA was relatively short and might be inadequate in most cases. Also, in breast reconstruction surgery, the internal thoracic vessels can be anastomosed with the vessels of the reconstruction flap to maintain blood flow. From this viewpoint, the lateral costal vessels could also be a candidate for anastomosis of the flap vessels during breast reconstruction surgery^10,11^.

Notably, recognizing the extent and course of lateral costal vessels via 3D-CT imaging preoperatively or prior to thoracentesis could help prevent unexpected injuries to the vessels (Fig. 5). Since the 4th or 5th ICS is commonly used for thoracotomy or as an access port during thoracoscopic surgery, lateral costal vessels extending further than the 4th ICS (23% in our study) could interfere with the incision. In our institute, we often insert the thoracoscope prior to making an incision for the access port or thoracotomy. Therefore, if we identify long lateral costal vessels extending further than the 4th ICS, we either avoid the vessels, or thoroughly coagulate or seal them prior to traversing the vessel, in order to avoid unexpected bleeding. However, dissection with electrocautery alone could be sufficient in cases with a small lateral costal vessel diameter. Preoperative recognition of the lateral costal vessels could be especially beneficial for uniportal thoracoscopic surgeries given that the single incision is often made at the 4th or 5th ICS of the anterior axillary line, close to the lateral costal vessels, and visualization of the vessels from lower thoracoscopic ports is not available.

In particular, we believe that the recognition of lateral costal vessels is important when performing thoracentesis for pneumothorax at the anterior or lateral chest wall, especially at the ‘triangle of safety’, which is an anatomical landmark that is usually recommended for needle insertion^12^. The lateral costal vessels may run at the center of this ‘triangle of safety’ (Fig. 5). Unawareness of this anatomical feature of lateral costal vessels could lead to unexpected injury to the vessels, thus causing hemothorax, similarly to injury of the intercostal artery.

One limitation of this study is that it was a retrospective analysis based on data from a single institution attending mostly to Japanese patients. Therefore, incidences and features might differ in other study populations. Moreover, our study mainly relied on visual assessment, which might differ from the incidence of lateral costal vessels detected by other methods, such as enhanced CT angiography. Also, the precise branching point of lateral costal vessels could not be analyzed from the intraoperative view alone, mainly due to the retrospective aspect of our analysis. We should be aware that although the LCA mostly branches from the ITA, it occasionally branches from more higher areas, i.e. the subclavian artery or the supreme intercostal artery^3^.

In conclusion, the LCA and LCV are relatively common yet not well defined vessels, compared to their counterparts, the internal thoracic vessels. The current study clarified the clinical, anatomical, and radiological features of lateral costal vessels based on intraoperative images in a large cohort. Our findings showed that the prevalence of lateral costal vessels was 19%, with male patients having higher rates, and extended further than the 2nd ICS in 74% of the cases. The lateral costal vessels can be clinically relevant in patients undergoing myocardial revascularization with ITA, pleurectomy, thoracic surgery, or thoracocentesis from the ‘triangle of safety’. Detection of the lateral costal vessels through 3D-CT, prior to thoracic surgery or thoracentesis could be beneficial to avoid unexpected injuries and bleeding.

## Methods

### Study population

This study included all patients over the age of 15 who underwent thoracic surgery at our institute between January 2016 and March 2020. Cases in which the lateral costal vessels could not be visualized were excluded due to patient-related or procedure-related reasons (i.e., lung adhesion, thick parietal pleura, or surgical procedure without sufficient intrathoracic view). Our study protocol was approved by the Medical Ethics Committee of Gunma University, Maebashi, Japan, which waived the requirement for informed consent (Approval Number: HS2020-179). The study was conducted according to the relevant guidelines and regulations.

### Analysis of clinical, anatomical, and radiological data

Charts were reviewed for age, sex, CT images, and 3D-CT images. Videos of surgical procedures using a thoracoscope (i.e. thoracoscopic surgery cases and thoracotomy/median sternotomy cases with thoracoscopic assistance) were analyzed for laterality, length, and distinct visual features of the lateral costal vessels. Three-dimensional CT images were reconstructed from conventional CT data.

### Statistical analysis

Summarized data are shown as medians with interquartile ranges for continuous variables and numbers and percentages for categorical variables. Significant differences were determined using the Pearson chi-square test and non-parametric Mann–Whitney U test. All statistical analyses were performed using the SPSS version 23.0 (IBM Corp., Armonk, NY), with statistical significance set at *p* < 0.05.

## Supplementary Information

Supplementary Figure 1.

Supplementary Figure 2.

Supplementary Legends.

## Abbreviations

3D-CT: Three-dimensional computed tomography
CABG: Coronary artery bypass grafting
ICG: Indocyanine green
ITA: Internal thoracic artery
LCA: Lateral costal artery
LCV: Lateral costal vein
ICS: Intercostal space
