# Supplementary figures and images for: Clinical and anatomical features of the lateral costal artery and vein
Source: Sci Rep. 2022 Jun 22;12:10589. doi: 10.1038/s41598-022-14318-3 (PMC9217911; doi:10.1038/s41598-022-14318-3)

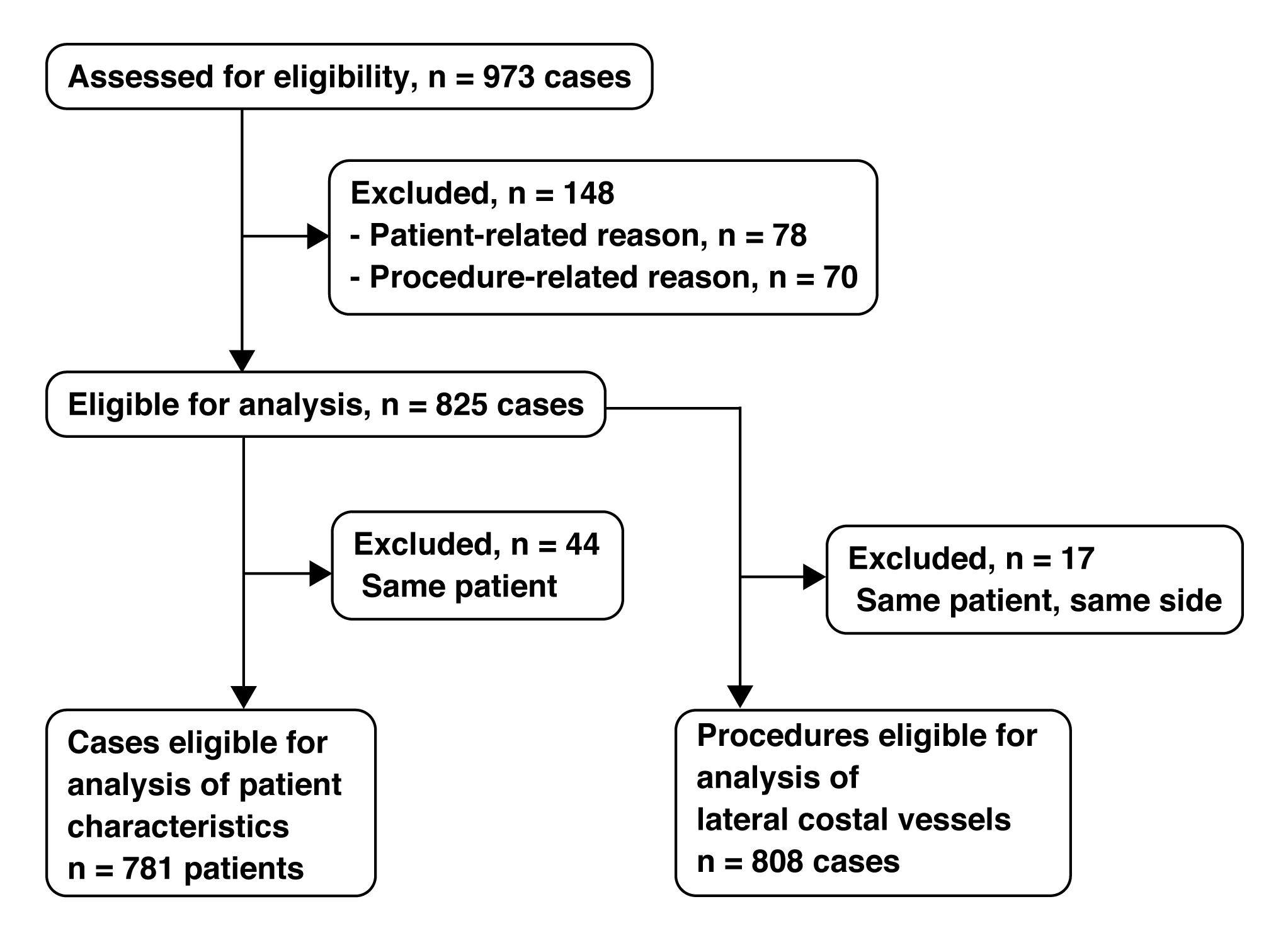

Supplement: Supplementary file 1 — Supplementary Figure 1. [file 41598_2022_14318_MOESM1_ESM.tif]

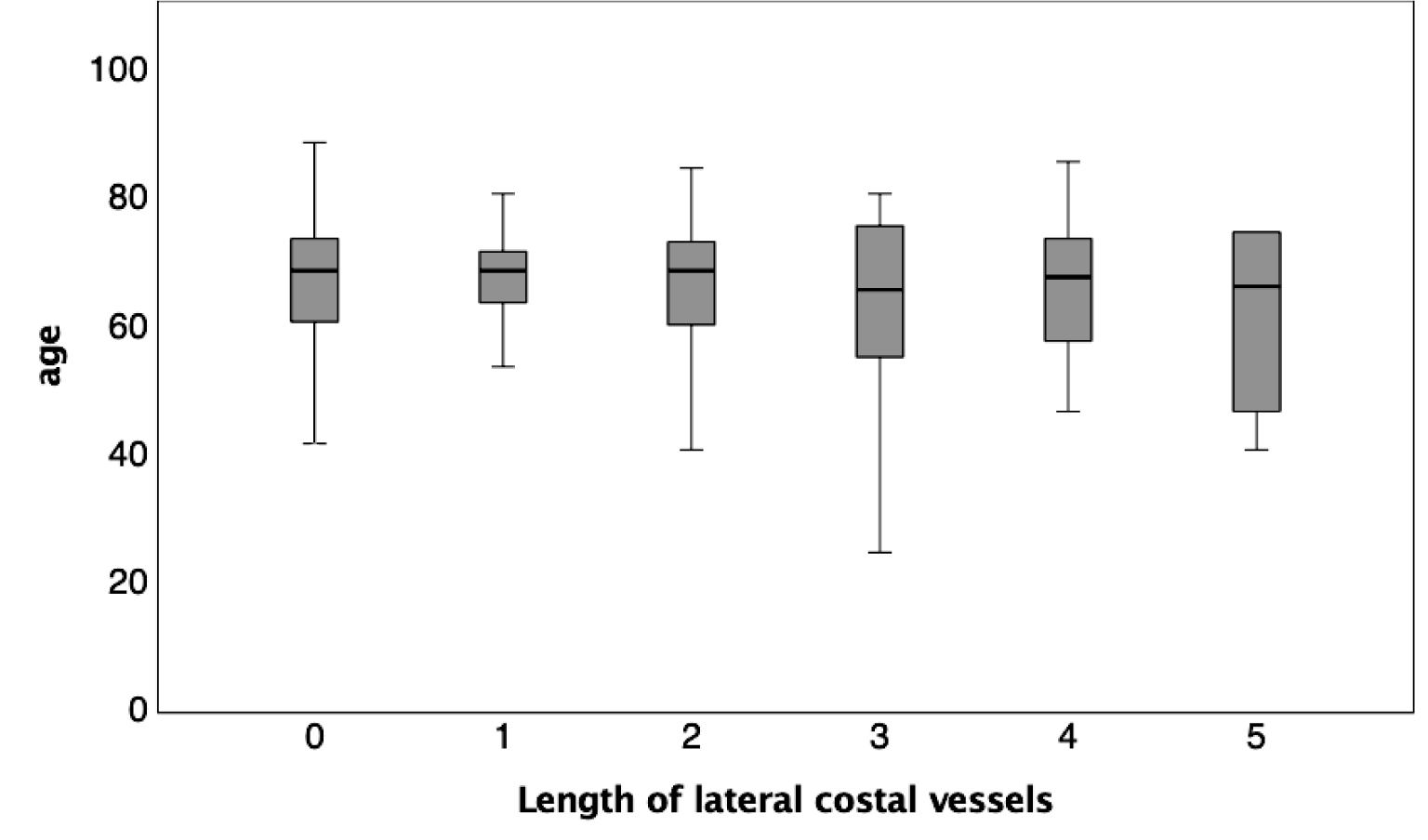

Supplement: Supplementary file 2 — Supplementary Figure 2. [file 41598_2022_14318_MOESM2_ESM.tif]
