# Supplementary material for: Clinical and anatomical features of the lateral costal artery and vein
Source: Sci Rep. 2022 Jun 22;12:10589. doi: 10.1038/s41598-022-14318-3 (PMC9217911; doi:10.1038/s41598-022-14318-3)
Supplement: Supplementary file 3 — Supplementary Legends. [file 41598_2022_14318_MOESM3_ESM.docx]

**Supplementary material.**

**Supplementary Figure 1. Flowchart of patient inclusion.**

A total of 973 surgical cases were initially included. Cases were excluded due to insufficient visual data for patient- (n = 78) and procedure-related reasons (n = 70). A total of 825 cases were analyzed. Some patients had multiple surgeries on the ipsilateral or contralateral side. Ultimately, 781 patients and 808 procedures were analyzed.

**Supplementary Figure 2. Correlation between age and length of lateral costal vessels.**

No significant difference in age was observed according to length of lateral costal vessels (p = 0.609). Y axis represents the intercostal space to which the vessels extend.
